# Punctate Inner Choroidopathy: A Systematic Review

**Published:** 2014

**Authors:** Joana Campos, António Campos, Sílvia Mendes, Arminda Neves, Diana Beselga, JP Castro Sousa

**Affiliations:** Ophthalmology Department, Leiria Hospital Center, Leiria, Portugal

**Keywords:** Punctate Inner Choroidopathy; Choroidal Neovascularization, Anti-VEGF Agents, Photodynamic Therapy

## Abstract

This article reviews clinically relevant data regarding punctate inner choroidopathy, mainly the various treatment options. Punctate inner choroidopathy is an uncommon, inflammatory, multifocal chorioretinopathy affecting mostly young myopic women. It is characterized by the presence of multiple, small, well-defined, yellow-white fundus lesions, in the absence of intraocular inflammation. We describe etiology, clinical findings and ancillary tests that help in the diagnosis and detection of complications. Treatment options that have been used to manage patients with PIC and CNV include immunosuppressants, corticoids, laser photocoagulation, photodynamic therapy, intravitreal anti-VEGF agents and submacular surgery.

## INTRODUCTION

The term punctate inner choroidopathy (PIC) was first used by Watzke, et al. (1) in 1984, to describe findings in a group of myopic patients with multifocal, well-circumscribed, small choroidal lesions after an infectious cause has been ruled out.

PIC is an infrequent ocular inflammatory disease, frequently affecting young myopic women. Patients present symptoms of loss of central visual acuity (VA), photopsia and scotomata. On fundoscopy, there are multiple, small, round, yellowish-white punctate lesions, in the absence of signs of intraocular inflammation (1, 2).

Spectral domain optical coherence tomography (SD-OCT) provides structural characteristics of PIC lesions (3). On fluorescein angiography (FA), the lesions fill and stain during the late phase. Autofluorescence (AF) images show hypofluorescence in cicatricial lesions and autofluorescence in active lesions (2).

PIC is usually a benign disease; however, severe visual loss can occur if complicated by choroidal neovascularization (CNV) and subretinal fibrosis (3). These complications usually occur within 1 year of presentation (4).

## METHODS

This systematic review describes PIC, using pertinent evidence available in the literature about epidemiology, clinical findings, ancillary tests, clinical course, complications and treatment.

Studies were identified by searching the electronic literature (Pubmed/MEDLINE) for reports published between 1984 and 2014, using the terms “punctate inner choroidopathy”, “choroidal neovascularization”, combined with “treatment”, “anti-VEGF agents” “optical coherence tomography”, “fluorescein angiography”, “indocyanine green angiography”.

Review articles and case reports were included. We also manually searched the reference lists of identified studies. Studies were assessed independently by two of the authors (JC and SM) and were not limited by language or country.

## ETIOLOGY

The underlying pathophysiology and etiology of the disorder are not entirely understood. Jampol, et al. (13) proposed that there is a familiar predisposition to autoimmune/inflammatory diseases, which leads to a response against antigens in the outer retina and inner choroid, triggered by an unknown agent. This process seems to be analogous to presumed ocular histoplasmosis syndrome (POHS), but further studies are required to analyze the genetic and environmental factors that contribute to the development of the disease. Some authors defend that PIC may be an independent inflammatory disease, while others suggest that it may be part of a spectrum of entities known as White Spot Syndromes of the Retina (13). 

Hirooka, et al. (14) quantified choroidal blood flow velocity at the macula using laser speckle flowgraphy in a patient with PIC treated with systemic corticosteroids. Choroidal blood flow decreased and choroidal thickness increased during the acute phase of PIC. Their results suggested that changes in the choroidal circulation related to inflammation may contribute to the pathogenesis of PIC and might involve a wider area in the posterior pole than the area of the PIC lesion.

## CLINICAL FINDINGS

In a recent study (15), 93% of 136 patients diagnosed with PIC were women, with a mean age at the initial visit of 32 years (range from 16 to 64) and mean spherical equivalent refraction of -4.6 diopters. The most common symptoms reported were photopsia and blurred vision. Gerstenblit, et al. (4) evaluated the demographic and clinical features of a sample of 77 persons with PIC. Of these, 90% were women and 85%were myopic. The median age was 30 years (range from 15 to 55). The most frequent symptom was scotomata in 91%. Other common symptoms were: blurred vision (86%), photopsia (73%), floaters (69%), photophobia (69%), metamorphopsia (65%), and loss of peripheral vision (26%). 

Essex, et al. (15) found that, at the initial visit, 47% of patients had unilateral disease and 53% had bilateral disease at baseline, 23% having uncomplicated disease and 77% presenting CNV. On the other hand, Brown, et al. (16) reported bilateral disease in 88% of patients with PIC. Considering VA loss, Essex, et al. (15) described a mean final VA of 5/10 or better in 178 eyes (66%) and less than 1/10 in 41 eyes (15%). The reason for loss of vision was recognized in 138 eyes: 119 eyes had CNV, 17 eyes had PIC lesions in the absence of CNV and 2 eyes had epiretinal membrane. Brown, et al. (16) studied a group of 30 eyes (16 patients), with a mean follow of 51 months. They found a mean VA of 5/10 or better in 77% (23 eyes) and 4/10 or worse in 23% (7 eyes), with 6 of these 7 eyes 1/10 or worse. Severe visual loss was mainly due to subfoveal CNV and fibrosis.

In our case report (17), anatomical improvement was achieved after treatment of CNV with intravitreal anti-VEGF, but there wasn’t prompt restoration of VA. There was a considerable improvement of VA one year after treatment, with a final VA of 5/10.

Biomicroscopy is characterized by the absence of ocular inflammation (vitritis, pars planitis, anterior chamber cells or anterior synechiae). Fundus examination reveals multiple, small (ranging from 100 to 300 μm in diameter), white or yellow, opaque round lesions, spread through the posterior pole or occasionally in a linear pattern (1). These lesions appear to be located at the level of the choroid and the retinal pigment epithelium (RPE) and can sometimes coalesce and form a serous retinal detachment (1, 3).

PIC is usually a benign disease, with favorable visual prognosis (5) and resolution of acute lesions in a few weeks. It frequently progress into atrophic chorioretinal scars. These scars can become pigmented after a few years, or regress, becoming clinically imperceptible (18).

## COMPLICATIONS

CNV and subretinal fibrosis are the most common and visual-threatening complications in PIC: rates of up to 69% and 56%, respectively, were reported in a series of 77 patients (4). In a smaller series of 12 patients, 75% had CNV (19).

The presence of lesions in the fellow eye of patients with unilateral PIC complicated with CNV seems to be a risk factor for development of CNV (15).

## ANCILLARY TESTS

*Optical Coherence Tomography (OCT)*

Channa, et al. (3) studied retinal changes on SD-OCT associated with PIC lesions. They found that the majority of the lesions (89%), regardless of clinical activity, presented involvement of the RPE, such as RPE elevation with sub-RPE signals and RPE discontinuity, overlying an apparently intact Bruch’s membrane.

Zarranz-Ventura, et al. (20) characterized a total of 90 PIC lesions, where 46.6% of them consisted of focal atrophy of the outer retina and RPE, 34.4% of sub-RPE hyperreflective deposits and 18.8% of focal RPE elevations with underlying hyporreflective space. It was suggested that this focal RPE elevation is seen in active phases, progressing later to focal atrophy of outer retinal layers or to sub-RPE hyperreflective deposits. Overall, SD-OCT can provide structural characteristics of PIC lesions, as well as information not detected clinically, such as early identification of recurrence of disease activity (3).

However, SD-OCT does not permit a detailed study about the extent of choroidal involvement (3).Enhanced depth imaging (EDI) technique can be used to study the choroid. Recently reports (14, 20) described its use in patients with PIC and found that choroidal thickness increases during the acute phase and decreases with regression of PIC. The use of OCT in PIC is also useful in the evaluation of treatment response in patients with CNV (20).

*Fluorescein Angiography*

Watzke, et al. (1) described that PIC lesions are hyperfluorescent in the early arterial phase, with staining in the arteriovenous phase. Olsen, et al. (5) studied5 patients (6 eyes) with CNV secondary to PIC and described their FA findings. CNV appeared as focal areas with an irregular, lacy network of neovascularization, with hyperfluorescence in the early phase and leakage in the late phase. With time, the new vessels formed a larger neovascular complex.

*Indocyanine Green Angiography*

Indocyanine green angiography (ICGA) findings show that PIC affects choriocapillaris and inner choroid (21). This exam shows mainly hypofluorescence in the early, middle and late phases, which probably corresponds to choroidal hypoperfusion. Tiffin, et al. (21) also described localized points of hyperfluorescence on the vessel walls that could suggest an associated choroidal vasculitic event.

## TREATMENT

Punctate inner choroidopathy is a relatively uncommon condition, with small published case series, so some features of the disease are not yet completely understood. The choice of the treatment for the PIC and CNV secondary to PIC has been limited by this lack of information. Many patients with PIC and no evidence of CNV do not need treatment, as the visual prognosis is good. However, treatment may be considered for those with inflammatory lesions (but no CNV) very close to fixation (18).

Recently, most research has been targeted at preventing visual impairment caused by the development of CNV. In one study, Gerstenblith, et al. (4) reported data from 77 patients diagnosed with PIC who were surveyed by the PIC Society. From those, 86% told they had received treatment: 60% with systemic corticosteroids, 22% with intraocular corticosteroids and 10% with periocular corticosteroids. In addition, approximately 14% reported treatment with at least one other immunosuppressive drug (mycophenolate mofetil, cyclosporine, methotrexate and azathioprine) during the course of the disease. Thirty-eight survey participants (49%) reported having received laser treatment (including PDT) (4).

*Corticosteroids*

Some authors have reported the use of systemic or periocular/intraocular steroids in PIC and in PIC-associated CNV (8, 22, 23). Levy, et al. (8) reported a case of a patient with juxtafoveal CNV due to PIC treated with oral corticosteroids. VA improved in 1 week, from 1/10 to 6.7/10, following treatment with oral prednisone 60 mg/day gradually tapered. Authors advocated that, although resolution of the PIC lesions may occur spontaneously, oral steroids help achieve more rapid recovery of VA. They also defend that steroids maybe used in cases with active CNV since they promote a reduction of the inflammatory and endothelial proliferative mechanisms.

Flaxel, et al. (22) used oral corticosteroids in patients with subfoveal CNV and PIC with visual symptoms, with good visual and anatomic outcome. The patients underwent a course of 1 mg/kg per day of oral prednisolone for 3-5 days, then tapered over 6-8 weeks. In this paper, they defended that oral systemic corticosteroids is a good option for healthy young patients with active subfoveal CNV secondary to PIC. However, there are some systemic and local adverse effects of corticosteroids that could justify avoiding this treatment in some patients (5).

*Immunomodulators*

Turkcuoglu, et al. (7) treated 8 patients with PIC, who had at least 2 recurrent episodes of increased activity, with mycophenolate mofetil. Authors concluded that this immunomodulatory therapy decreased frequency of attacks in recurrent PIC.

*Intravitreal Anti-VEGF*

In the last years, several studies have been published considering the use of intravitreal anti-VEGF for treating CNV in PIC (Table 1).

Chan, et al. (12) reported 4 cases of CNV due to PIC treated with 3 monthly injections of intravitreal bevacizumab. After 6 months of follow-up, all patients improved and none had VA worse than 5/10. All cases showed no angiographic leakage at months 3 and 6. In 2010, Leung, et al. (24) reported a case of PIC complicated by CNV treated with a single injection of intravitreal ranibizumab. There was improvement in VA, with no recurrence of disease activity at 8 months. Menezo, et al. (25) described results of 10 patients with CNV secondary to PIC which were treated with intravitreal ranibizumab (average of 1.9 injections). Previous therapies included PDT, sub-tenon triamcinolone, oral prednisolone and intravitreal triamcinolone. Nine eyes maintained or improved vision, after treatment. No ocular or systemic complications were observed.

Mangat, et al. (11) described successful cases of 4 patients with CNV secondary to PIC treated solely with anti-VEGF in 3 cases and in combination with PDT in the other. Authors proposed that anti-VEGF should be the first line of treatment due to good results without serious complications, although frequent follow-up for a long period is recommended to detect recurrences. They also emphasized that some patients **w**ith PIC-related CNV may be less responsive to anti-VEGF, requiring other treatment options.

Rouvas, et al. (26) evaluated the effect of intravitreal injections of ranibizumab on VA and central foveal thickness in 15 patients with CNV associated with various ocular inflammatory clinical entities, 5 of them with PIC. Repeated intravitreal injections were performed when persistent or recurrent fluid on OCT and/or signs of active CNV on angiography were present. The mean follow-up time was 70.4 ± 24 weeks. Their study supports the promising results of intravitreal ranibizumab in VA improvement and decrease in macular thickness in patients with inflammatory CNV.

Zhang, et al. (27) evaluated the efficacy and safety of intravitreal bevacizumab as primary treatment of CNV secondary to PIC in 12 patients (12 eyes). All eyes had stable or improved vision. Mean best-corrected VA improved from 3.2/10at baseline to 5.9/10 at 12 months of follow-up. Mean central retinal thickness decreased from 333 μm to 241 μm. No systemic or ocular side effects were observed.

We published, in 2013, a case report of a 29-year-old caucasian male with PIC complicated by CNV (17). A single intravitreal injection of bevacizumab 1.25 mg/0.05 ml was given, with anatomic and visual improvement. Even though some authors used 3 monthly injections of anti-VEGF (12, 28), we treated with only 1 injection and the patient showed no need of additional doses. There was no recurrence after 2 years of follow up.

In 2014, Jiménez, et al. (28) reported a case report of a female patient with CNV due to PIC, treated with intravitreal injections of ranibizumab in a monthly administration up to 3 injections. One month after the third injection, VA reached 10/10 and OCT revealed no intra nor subretinalfluid. There was no recurrence during the following year.

**Table 1:** Studies Considering the Use of Intravitreal Anti-VEGF for Treating CNV in PIC

**Author**	**Year**	**N (Patients)**	**N (Eyes)**	**Treatment**	**Dose**
**Chan, et al. (12)**	2007	4	4	Bevacizumab	1.25 mg/0.05 ml
**Leung, et al. (24)**	2010	1	1	Ranibizumab	0.5 mg/0.1 ml
**Menezo, et al.(25)**	2010	10	10	Ranibizumab	0.5 mg/0.1 ml
**Mangat, et al. (11)**	2011	4	4	Ranibizumab/Bevacizumab	0.5 mg/0.1 ml / 1.25 mg/0.05 ml
**Rouvas, et al. (26)**	2011	5	5	Ranibizumab	0.5 mg/0.1 ml
**Zhang, et al. (27)**	2012	12	12	Bevacizumab	1.25 mg/0.05 ml
**Campos, et al. (17)**	2013	1	1	Bevacizumab	1.25 mg/0.05 ml
**Jiménez, et al. (28)**	2014	1	1	Ranibizumab	0.5 mg/0.1 ml

**IVT = Intravitreal**

*Laser Photocoagulation*

Brown, et al. (16) reported 2 cases with extrafoveal CNV due to PIC, treated with laser photocoagulation. Both patients had final VA of 5/10 or better, but initial VA was not stated in the paper.

There is an increased risk of developing CNV after argon laser due to aggression and rupture of the Bruch’s membrane. Additionally, laser scars enlarge with time and the fovea may be later involved.

*Photodynamic Therapy*

Postelmans, et al. (29) analyzed cases of 7 patients (7 eyes) treated only with PDT. Mean VA increased from 4.5/10 to 7.5/10, after a mean follow-up of 22 months and a mean number of 2.5 (range from 1 to 4) PDT sessions. VA remained stable or improved in 6 of the 7 eyes and decreased in only1 eye. No serious systemic or ocular side effects related to PDT were observed.

Coco, et al. (30) performed a retrospective analysis of 8 patients (8 eyes) with PIC treated with PDT (mean of 3.3 treatments per patient), followed for a mean of 22.7 months. They concluded that PIC seems to have relatively higher rates of CNV recurrence after PDT compared to other inflammatory or idiopathic diseases; however, this rate seems to be lower than that after submacular surgery (5). In this study, 2 patients suffered CNV recurrences more than15 months after initial PDT treatment and required additional PDT sessions. In this way, authors hypothesized that there is a trend for CNV in PIC to recur a long time after PDT treatment.

Brouzas, et al. (6) reported on along-term follow-up study (average of 105 months) CNV cases secondary to PIC: 4 patients (4 eyes) treated with PDT and4 patients (5 eyes) left without treatment. Improvement of VA was observed in 6 eyes, including all the treated eyes and 2 of the 5 untreated eyes. The size of the lesions, including CNV and subretinal fibrosis, increased in all cases left untreated and remained stable in all cases treated with PDT. The authors concluded that, even though the untreated CNV due to PIC is slowly progressive, treatment with PDT appears to help in maintaining VA and stabilizing the progression of CNV and fibrosis.

*Submacular Surgery*

In 1996, Olsen, et al. (5) reported 5 patients (6 eyes) with PIC and subfoveal CNV treated with submacular surgery. Every patient improved VA after surgery. The mean follow-up was 14 months. Nevertheless, recurrences were common (66.7%) and resulted in a significant decrease of VA. They concluded that submacular surgery may be a successful treatment for the CNV associated with PIC in selected patients, depending on the timing and location of CNV (they did not recommend surgical treatment of extrafoveal CNV). The high rate of recurrence observed and the additional costs and risks of surgery can also influence the treatment choice.

## CONCLUSION

PIC is an inflammatory disease affecting choroid and RPE. In general, the prognosis is good and some cases do not need treatment. Nevertheless, there are severe cases, complicated with CNV, that require treatment and can lead to visual impairment.

There is a lack of studies with a large number of patients that might clarify the cause, precipitating factors and pathophysiology. This information would be useful to define the more appropriate treatment.

Intravitreal anti-VEGF treatment appears to be a good choice as the first line of treatment in CNV secondary to PIC. However, there is a need for further prospective studies with larger samples to confirm safety, efficacy and the best treatment regimen.
